# Nobiletin Ameliorates Alzheimer’s Disease Pathology by Reducing Oxidative Stress and Neuroinflammation Through the AMPK/SIRT1/PGC-1*α* and PI3K/Akt–CREB–BDNF Pathways in 5XFAD Mice

**DOI:** 10.3390/biomedicines14030561

**Published:** 2026-02-28

**Authors:** Hana Baek, Miey Park, Hae-Jeung Lee

**Affiliations:** 1 Department of Food and Nutrition, College of BioNano Technology, Gachon University, Seongnam-si 13120, Gyeonggi-do, Republic of Korea; ruru456123@naver.com; 2 Institute for Aging and Clinical Nutrition Research, Gachon University, Seongnam-si 13120, Gyeonggi-do, Republic of Korea; 3 Department of Health Sciences and Technology, Gachon Advanced Institute for Health Science and Technology (GAIHST), Gachon University, Incheon 21999, Gyeonggi-do, Republic of Korea

**Keywords:** Alzheimer’s disease, nobiletin, oxidative stress, neuroinflammation, AMPK/SIRT1/PGC-1*α*, PI3K/Akt–CREB–BDNF, synaptic plasticity

## Abstract

**Background/Objectives:** Alzheimer’s disease (AD) involves amyloid-*β* (A*β*) deposition, oxidative stress, and neuroinflammation, leading to cognitive decline. Nobiletin, a citrus-derived polymethoxylated flavonoid, exerts antioxidant and anti-inflammatory effects. This study explored its neuroprotective mechanisms in the 5XFAD mouse model. **Methods:** Male 5XFAD and C57BL/6J mice received oral nobiletin (20 or 40 mg/kg/d) for 4 weeks. Cognitive function was assessed by the Y-maze test. Amyloid-*β* burden was quantified by Congo red staining and ELISA. Serum cytokine levels and antioxidant enzyme activities were measured by ELISA. Western blotting and RT-PCR were used to assess proteins and genes related to amyloidogenesis, inflammation (TLR4/MyD88/NF-*κ*B), mitochondrial biogenesis (AMPK/SIRT1/PGC-1*α*), and synaptic plasticity (PI3K/Akt–CREB–BDNF). **Results:** Nobiletin improved working memory, reduced amyloid-*β*40/42 deposition, and downregulated APP, BACE1, and PS1 expression, while enhancing ADAM10 expression. It lowered serum IL-6, IL-1*β*, and TNF-*α*, increased SOD, CAT, and GPx activities, and suppressed TLR4/MyD88/NF-*κ*B signaling. Furthermore, it activated AMPK/SIRT1/PGC-1*α* and NRF2 pathways, enhancing antioxidant defenses, and promoted PI3K/Akt–CREB–BDNF signaling, increasing PSD95 and synaptophysin. **Conclusions:** Nobiletin exerts strong neuroprotective and antioxidant effects by targeting multiple signaling cascades, mitigating amyloid pathology and neuroinflammation, and improving synaptic plasticity. It represents a promising therapeutic agent against AD.

## 1. Introduction

Alzheimer’s disease (AD) is one of the most prevalent neurodegenerative diseases and significantly affects cognitive abilities, daily functioning, memory, and judgment in humans [1,2,3]. Despite extensive studies, the exact pathophysiological mechanisms underlying AD remain unclear. Recent studies have focused on unraveling the complex features of this disease and exploring various therapeutic approaches to mitigate its impact.

A hallmark pathological feature of AD is the accumulation of amyloid-*β* (A*β*) plaques and neurofibrillary tangles (NFTs) in the brain. Amyloid precursor protein (APP), which is ubiquitously expressed in various cell types, undergoes proteolytic processing via amyloidogenic and non-amyloidogenic pathways. In the non-amyloidogenic pathway, APP is cleaved by *α*- and λ-secretases into soluble A*β* fragments, which are non-toxic. In contrast, the amyloidogenic pathway involves cleavage by *β*-secretase, resulting in the production of insoluble A*β* peptides [4]. Among these, the A*β*40 and A*β*42 isoforms are particularly neurotoxic [5]. Additionally, the microtubule-associated protein tau, which is essential for maintaining neuronal structure and facilitating nutrient transport, is hyperphosphorylated in AD, leading to the formation of neurofibrillary tangles [6,7].

Oxidative stress, which is characterized by the accumulation of free radicals, is essential to AD progression. Antioxidants counteract oxidative stress, thereby reducing cellular damage in the brain and potentially slowing the progression of AD [8,9]. Similarly, anti-inflammatory mechanisms are crucial for modulating AD onset and progression by regulating immune cell activity, particularly microglial activity, and suppressing neuroinflammation [10,11,12]. Synaptic function is vital for maintaining brain health. Compounds with antioxidant and anti-inflammatory properties enhance synaptic function by reducing inflammation and protecting neuronal integrity [13,14].

Nobiletin, a polymethoxylated flavonoid abundant in citrus fruits, has multiple health benefits, including anti-inflammatory effects in osteoarthritis, regulation of insulin resistance in diabetes, and modulation of apoptosis in cancer cells [15,16,17]. Notably, nobiletin exhibits neuroprotective effects against neurodegenerative diseases by inhibiting amyloid accumulation and reducing neuroinflammation [18,19]. Although previous studies have highlighted nobiletin’s role in ameliorating cognitive impairment and improving AD symptoms, its effects on synaptic plasticity via antioxidant and anti-inflammatory pathways remain poorly understood.

In this study, we investigated the therapeutic potential of nobiletin to enhance synaptic plasticity via its antioxidant and anti-inflammatory effects in a 5XFAD transgenic mouse model of AD. We hypothesized that nobiletin’s multifaceted biological activities could synergistically improve synaptic function and cognitive outcomes in AD by mitigating oxidative stress and neuroinflammation. This investigation deepened our understanding of nobiletin’s role in synaptic plasticity and its broader therapeutic implications in AD. This research aims to deepen knowledge of nobiletin’s multifaceted role in combating AD pathology and to explore its therapeutic potential as a candidate for managing neurodegenerative disorders.

## 2. Materials and Methods

### 2.1. Materials

Nobiletin was purchased from ChemFaces Biochemical Co. (Wuhan, China). Antibodies against phospho-AMP-activated protein kinase (pAMPK), AMPK, nuclear factor erythroid-2-related factor 2 (NRF2), heme oxygenase-1 (HO-1), superoxide dismutase 2 (SOD2), phospho-phosphoinositide 3-kinase (pPI3K), PI3K, phospho-protein kinase B (pAkt), Akt, phospho-cAMP response element-binding protein (pCREB), CREB, postsynaptic density protein-95 (PSD95), and *β*-actin were obtained from Cell Signaling Technology (Danvers, MA, USA). The anti-APP, *β*-secretase (BACE1), presenilin-1 (PS1), *α*-secretase (ADAM10), and sirtuin 1 (SIRT1) were purchased from Abcam (Cambridge, UK). The anti-SYP antibody was obtained from Invitrogen (Carlsbad, CA, USA). The anti-peroxisome proliferator-activated receptor gamma coactivator 1-alpha (PGC1*α*) antibody was obtained from Bioss (Woburn, MA, USA). The anti-brain-derived neurotrophic factor (BDNF) antibody was obtained from ABclonal (Woburn, MA, USA). The interleukin (IL)-6, IL-1*β*, and tumor necrosis factor alpha (TNF-*α*) enzyme-linked immunosorbent assay (ELISA) kits were obtained from R&D Systems (Minneapolis, MN, USA). SOD and catalase (CAT) ELISA kits were purchased from LS Bio (Lynnwood, NJ, USA). The glutathione peroxidase (GSH-Px) ELISA kit was purchased from Novus Biologicals (Centennial, CO, USA).

### 2.2. Animals and Diets

Five-week-old male 5XFAD transgenic (B6SJL-Tg [APPSwFlLon, PSEN1*M146L*L286V] 6799Vas/Mmjax) and C57BL/6 J mice were purchased from Jackson Laboratory (West Grove, CA, USA). Genotyping was confirmed by PCR using APP and PSEN1 primers, as described by Jackson. Mice underwent a 17-week acclimation period under controlled conditions (50–60% humidity at 20–25 °C). After acclimation, the mice were divided into four groups: C57BL/6 J mice (NC, *n* = 10), 5XFAD mice administered 0.5% carboxymethyl cellulose (CMC; 5XFAD, *n* = 10), 5XFAD mice treated with 20 mg/kg/d nobiletin dissolved in 0.5% CMC (5XFAD_NOB20, *n* = 10), and 5XFAD mice treated with 40 mg/kg/d nobiletin dissolved in 0.5% CMC (5XFAD_NOB40, *n* = 10) for 4 weeks. The mice were monitored for 21 weeks with unrestricted access to food and water. Food intake and body weight were recorded weekly during oral administration. The 5XFAD transgenic mouse model is known to exhibit an aggressive Alzheimer’s-like pathology, with amyloid-*β* (A*β*) deposition beginning as early as two months of age and substantial plaque accumulation and neuronal loss evident by five months. Therefore, the present study, which utilized 5-month-old 5XFAD mice, was designed to evaluate the therapeutic rather than preventive effects of nobiletin after pathological A*β* accumulation had been established [20,21,22]. All animal experiments were conducted at Eulji University under a collaborative agreement with Gachon University, where the researchers are affiliated.

### 2.3. Microscopy Congo Red Staining

After 21 weeks, the mice were fasted for 12 h and killed using CO_2_ asphyxiation. First, perfusion was performed using ice-cold phosphate-buffered saline through the left ventricle at a flow rate of 10 mL/min until the effluent was clear and free of blood. They were then perfused with 4% paraformaldehyde at the same flow rate for 10–15 min. After perfusion, the brains were removed and fixed in 10% formalin. Congo red staining was performed to detect A*β* plaques in brain tissue. All images were acquired at 40× magnification using a Leica microscope (Leica, Wetzlar, Germany). The same threshold parameters were used for all groups to avoid bias. Regions of interest were manually defined for the cortex and hippocampus matched brain areas. All image analyses were conducted in a blinded manner, with the investigator unaware of group allocation to minimize bias. The number of stained A*β* plaques in hippocampal and cortical regions was quantified using ImageJ (1.x, Bethesda, MD, USA).

### 2.4. ELISA

To quantify A*β*1-40 or A*β*1-42 levels, hippocampal and cortical tissue samples were homogenized in cold 20 mM Tris buffer (for soluble fractions) or 5 M guanidine HCl/50 mM Tris-HCl buffer (for insoluble fractions). After homogenization, total protein concentration was determined using a BCA protein assay kit (Thermo Fisher Scientific, Rockford, IL, USA). Soluble and insoluble A*β*1-40 and A*β*1-42 levels in brain tissue were normalized to total protein content and expressed as pg/mg protein. The levels of soluble and insoluble A*β*1-40 and A*β*1-42 in serum were assessed using ELISA kits (Invitrogen, Thermo Fisher Scientific, Waltham, MA, USA). Serum levels of IL-6, IL-1*β*, TNF*α*, SOD, CAT, and GPx were determined according to the manufacturers’ instructions and expressed as pg/mL or ng/mL.

### 2.5. Y-Maze Test

The Y-maze test was performed at 3 and 4 weeks after oral administration commenced. After oral administration on the test day, the mice were allowed to stabilize for over 3 h. The Y-maze had three identical arms (35 cm long, 3 cm wide, and 15 cm high) angled at 120°. Before testing began, the three arms were randomly assigned as arm A, B, or C. At the beginning of the test, the mice were placed at the end of arm A and allowed to freely explore the maze for 8 min. An entry was recorded when the entire body of the mouse passed through one-quarter of an arm. Spontaneous alternation was defined as choosing three different arms consecutively during the test. The percentage of alternations was calculated as the number of spontaneous alternations divided by the total number of entries minus two.

### 2.6. Immunoblot Analysis

Total protein was extracted from the hippocampus and cortex for protein expression analysis, with four mice per group. Hippocampal and cortical tissues were homogenized in a lysis buffer containing protease and phosphatase inhibitors (iNtRON Biotechnology, Seongnam-si, Republic of Korea). The homogenates were centrifuged at 13,000 rpm for 10 min at 4 °C to obtain the supernatant. The extracted proteins (40 μg) were separated by electrophoresis on polyacrylamide gels and transferred to polyvinylidene fluoride membranes (Bio-Rad Laboratories, Hercules, CA, USA). The membranes were blocked with 5% skim milk (BD Biosciences, Franklin Lakes, NJ, USA) at room temperature and then incubated overnight at 4 °C with the following primary antibodies: APP (1:1000, ab32136), BACE1 (1:1000, ab183612), PS-1 (1:500, ab15458), ADAM10 (1:1000, ab124695), pAMPK (1:1000, 2535S), AMPK (1:1000, 2532S), SIRT1 (1:1000, ab110304), PGC1α (1:1000, bs-7535R), NRF2 (1:500, 12721S), HO-1 (1:1000, 43966S), SOD2 (1:1000, 13141S), pPI3K (1:1000, 4228S), PI3K (1:1000, 4292S), pAkt (1:1000, 4060S), Akt (1:1000, 9272S), pCREB (1:500, 9198S), CREB (1:500, 9197S), BDNF (1:1000, A1307), PSD95 (1:1000, 3450S), synaptophysin (SP11, 1:1000, MA5-14532), and β-actin (1:1000, 3700S). After overnight incubation, the membranes were incubated with secondary antibodies for 1 h at room temperature. Protein bands were detected using a Miracle-Star detection system (iNtRON Biotechnology, Seongnam-si, Republic of Korea) and imaged using a Quant LAS 500 system (GE Healthcare Bio-Sciences AB, Uppsala, Sweden). The band intensities were then quantified using ImageQuant software (version 10.2, Cytiva, Uppsala, Sweden), and the expression of each target protein was normalized to its corresponding *β*-actin loading control on the same blot. Although β-actin was stable across groups, future studies using multiple reference genes would further strengthen normalization. When multiple proteins within the same signaling pathway were analyzed, membranes were reused after mild stripping with a stripping buffer to minimize protein loss, then reblocked and incubated with antibodies against additional target proteins.

### 2.7. RNA Preparation and Real-Time PCR (RT-PCR) Analysis

Hippocampal and cortical tissues were homogenized in lysis buffer, and total RNA was extracted using an RNA extraction kit (iNtRON Biotechnology, Seongnam-si, Republic of Korea). The extracted total RNA (50 ng) was reverse transcribed into cDNA using a PCR Thermal Cycler Dice Touch (TaKaRa Bio, Shiga, Japan). Reverse transcribed cDNA was mixed with primers and TB Green^®^Premix Ex Taq™ II (TaKaRa Bio, Shiga, Japan), and RT-PCR was performed on an ABI Quant Studio 3 PCR system (Applied Biosystems, Waltham, MA, USA). *β*-actin was used as an internal reference. The PCR primer sequences were as follows: Toll-like receptor 4 (TLR4): 5′-AGCTTCTCCAATTTTTCAGAACTTC-3′ (forward) and 5′-TGAGAGGTGGTGTAAGCCATGC-3′ (reverse); myeloid differentiation primary response 88 (MyD88): 5′-CTAGGACAAACGCCGGAACT-3′ (forward) and 5′-ATTAGCTCGCTGGCAATGGA-3′ (reverse); nuclear factor kappa b: 5′-GCTGCCAAAGAAGGACACGACA-3′ (forward) and 5′-GGCAGGCTATTGCTCATCA CAG-3′ (reverse); NLR family pyrin domain containing 3 (NLRP3): 5′-TCACAACTCGCCCAAGGAGGAA-3′ (forward) and 5′-AAGAGACCACGGCAGAAGCTAG-3′ (reverse); cluster of differentiation 86 (CD86): 5′-ACGATGGACCCCAGATGCACCA-3′ (forward) and, 5′-GCGTCTCCACGGAAACAGCA-3′ (reverse); cyclooxygenase-2 (COX-2): 5′-AGAAGGAAATGGCTGCAGAA-3′ (forward) and 5′-GCTCGGGCTTCCAGTATTGAG-3′ (reverse); inducible nitric oxide synthase (iNOS): 5′-TTCCAGAATCCCTGGACAAG-3′ (forward) and 5′-TGGTCAAACTCTTGGGGTTC-3′ (reverse); triggering receptor expressed on myeloid cells 2 TREM2: 5′-TGGGACCTTCCCACCAGTT-3′ (forward) and 5′-GTGGTGTTGAGGGCTTGG-3′ (reverse); Fndc5: 5′-GGACTCTTGGAAAACACCACTG-3′ (forward) and 5′-TCCACACAGATGATCTCACCAC-3′ (reverse); IL-10: 5′-GCTCAGCACTGCTATGCTG-3′ (forward) and 5′-GCAGTATGTTGTCCAGCTGG-3′ (reverse); cluster of differentiation 206 (CD206): 5′-TCAGCTATTGGACGCGAGGCA-3′ (forward) and 5′-TCCGGGTTGCAAGTTGCCGT-3′ (reverse); arginase 1 (Arg-1): 5′-CTTGCGAGACGTAGACCCTG-3′ (forward) and 5′-TCCATCACCTTGCCAATCCC-3′ (reverse); *β*-actin: 5′-CCACAGCTGAGAGGAAATC-3′ (forward) and 5′-AAAGGAAGCTGGAAAAGAGC-3′ (reverse).

### 2.8. Statistical Analysis

Statistical analyses were performed using the GraphPad Prism 10.2.2 software (La Jolla, CA, USA). Data are presented as mean ± standard error (SE). One-way analysis of variance was used for statistical comparisons, followed by Dunnett’s test to determine specific group differences. Statistical significance was represented as * *p* < 0.05, ** *p* < 0.01, *** *p* < 0.001, and **** *p* < 0.0001.

## 3. Results

### 3.1. Nobiletin Enhances Learning and Memory in a 5XFAD Mouse Model of AD

**Figure 1:**
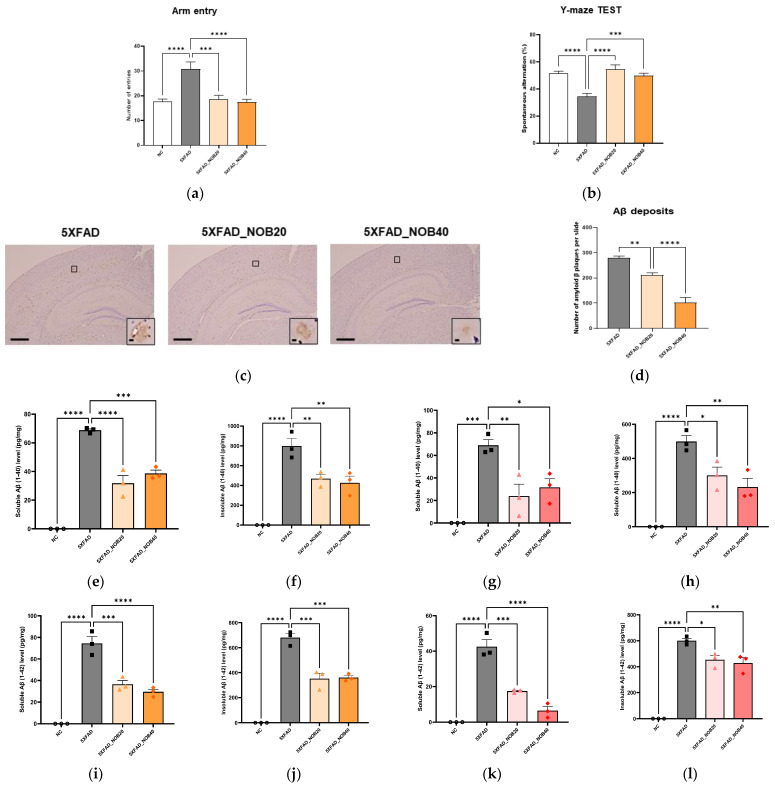
Nobiletin Enhances Cognitive Performance and Reduces Amyloid Plaque Burden in 5XFAD Mice. (**a**) Number of arm entries and (**b**) spontaneous alternation rate in the Y-maze test indicating improved working memory following nobiletin administration. Data represent the Y-maze tests performed at week 4. (**c**,**d**) Congo red staining and quantification showing reduced amyloid plaque accumulation in the cortex and hippocampus. (**e**–**l**) ELISA quantification of soluble and insoluble A*β*40 and A*β*42 in both cortical and hippocampal tissues. Mice were divided into four groups: non-transgenic control (NC), 5XFAD vehicle-treated (5XFAD), and 5XFAD mice treated orally with nobiletin at 20 mg/kg/d (5XFAD_NOB20) or 40 mg/kg/d (5XFAD_NOB40). The Y-maze test was conducted to calculate the alternation ratio, defined as the number of actual alternations divided by the maximum possible alternation (*n* = 7). Extracellular A*β* accumulation was analyzed using Congo red staining (*n* = 3). Scale bar is 100 μm. Statistical significance was represented as * *p* < 0.05, ** *p* < 0.01, *** *p* < 0.001, and **** *p* < 0.0001; bars represent mean ± SE.

The working memory of mice was assessed using the Y-maze test, which quantifies arm entries and spontaneous alternation rate as indicators of spatial memory performance (Kraeuter et al., 2019) [23]. The results demonstrated that the 5XFAD group had the highest number of arm entries (Figure 1a, *p* < 0.0001). However, the spontaneous alternation rate was significantly lower in the 5XFAD group than in the Normal control (NC) group (Figure 1b, *p* < 0.0001). Notably, treatment with 20 (5XFAD_NOB20) and 40 mg/kg (5XFAD_NOB40) nobiletin significantly improved the spontaneous alternation rate (Figure 1b). These findings suggest that nobiletin effectively enhances working memory function in a 5XFAD transgenic mouse model of AD.

### 3.2. Nobiletin Reduces Aβ Plaques in the Brains of 5XFAD Mice

To evaluate the impact of nobiletin on A*β* pathology, we quantified A*β* plaques in the brains of 5XFAD mice using Congo red staining. The analysis revealed that the 5XFAD_NOB20 and 5XFAD_NOB40 groups exhibited reduced A*β* levels compared to the untreated 5XFAD group, with the 5XFAD_NOB40 group demonstrating a significant reduction (Figure 1c,d). Furthermore, the concentrations of both soluble and insoluble A*β*1-40 and A*β*1-42, the predominant components of amyloid plaques in AD pathology, were measured in the cortical and hippocampal regions. The results indicated that the levels of soluble and insoluble A*β*1-40 and A*β*1-42 were significantly elevated in the 5XFAD group compared to those in the nobiletin-treated groups (Figure 1e–l). Notably, a pronounced decrease in A*β* levels was observed following nobiletin administration, demonstrating that nobiletin effectively reduces A*β* plaque burden in the brains of 5XFAD mice and suggesting its potential therapeutic efficacy in mitigating amyloid pathology in AD.

### 3.3. Nobiletin Reduces Systemic Inflammatory Responses and Oxidative Stress in 5XFAD Mice

**Figure 2:**
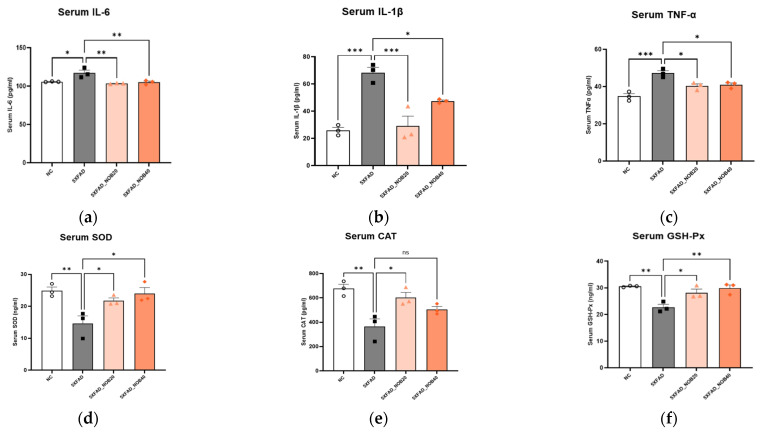
Nobiletin Reduces Neuroinflammatory Responses and Oxidative Stress in 5XFAD Mice serum. (**a**) Interleukin (IL)-6, (**b**) interleukin (IL)-1*β*, (**c**) tumor necrosis factor-*α* (TNF-*α*), (**d**) superoxide dismutase (SOD), (**e**) catalase (CAT), and (**f**) glutathione peroxidase (GSH-Px). Mice were divided into four groups: non-transgenic control (NC), 5XFAD vehicle-treated (5XFAD), and 5XFAD mice treated orally with nobiletin at 20 mg/kg/d (5XFAD_NOB20) or 40 mg/kg/d (5XFAD_NOB40). Statistical significance was represented as * *p* < 0.05, ** *p* < 0.01, and *** *p* < 0.001; bars represent mean ± SE.

Serum levels of IL-6, IL-1*β*, and TNF-*α* were significantly higher in the 5XFAD group than in the NC group and decreased after nobiletin administration (Figure 2a–c). SOD, CAT, and GPx are antioxidant enzymes that protect against oxidative stress-induced neuronal damage [24]. The serum levels of SOD, CAT, and GPx significantly decreased in the 5XFAD group compared to those in the NC group but increased in the 5XFAD_NOB20 group; SOD and GPx levels also increased in the 5XFAD_NOB40 group (Figure 2d–f). These findings demonstrate that nobiletin significantly decreased serum pro-inflammatory cytokines and enhanced systemic antioxidant enzyme activities.

### 3.4. Nobiletin Alleviates AD Pathology Mechanisms

**Figure 3:**
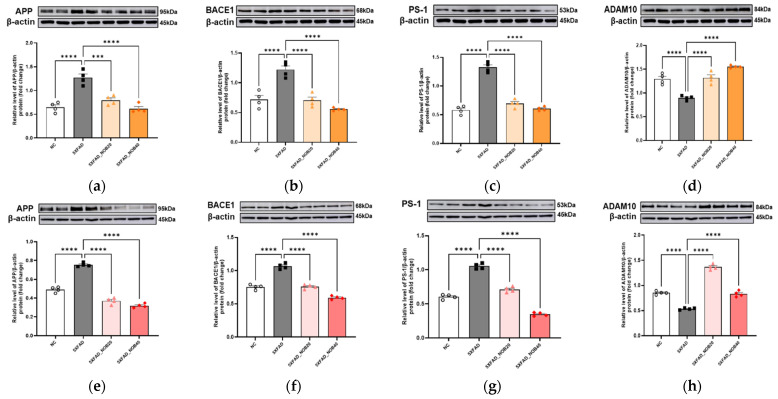
Nobiletin Alleviates AD Pathology Mechanisms in 5XFAD Mice. (Protein expressions in cortical (**a**–**d**) and hippocampal (**e**–**h**) regions were analyzed by Western blotting using biological replicates from individual mice (*n* = 4). Expression of amyloid precursor protein (APP, (**a**,**e**)); *β*-secretase (BACE1, (**b**,**f**)); presenilin-1 (PS1, (**c**,**g**)); and *α*-secretase (ADAM10, (**d**,**h**)). Mice were divided into four groups: non-transgenic control (NC), 5XFAD vehicle-treated (5XFAD), and 5XFAD mice treated orally with nobiletin at 20 mg/kg/d (5XFAD_NOB20) or 40 mg/kg/d (5XFAD_NOB40). The *β*-actin blots were generated on the same membrane after stripping and reprobing with different primary antibodies. Statistical significance was represented as *** *p* < 0.001, and **** *p* < 0.0001; bars represent mean ± SE.

We observed significant upregulation of APP, BACE1, and PS1 protein levels, whereas ADAM10 expression was significantly reduced in the cortical and hippocampal regions of 5XFAD mice compared with the NC group (*p* < 0.0001). However, in the 5XFAD_NOB20 and 5XFAD_NOB40 groups, the protein expression of APP (Figure 3a,e, *p* < 0.0001), BACE1 (Figure 3b,f, *p* < 0.0001), and PS1 (Figure 3c,g, *p* < 0.0001) was significantly downregulated compared to that in the 5XFAD group. These findings suggest that nobiletin administration inhibits the amyloidogenic pathway by decreasing the expression of key proteins involved in A*β* production. In contrast, ADAM10 protein (Figure 3d,h, *p* < 0.0001) expression showed an opposite trend, increasing in the nobiletin-treated groups compared with the 5XFAD group.

### 3.5. Nobiletin Alleviates Neuroinflammation in the Cortical and Hippocampal Regions of 5XFAD Mice

#### 3.5.1. Microglial Activation-Related Markers

**Figure 4:**
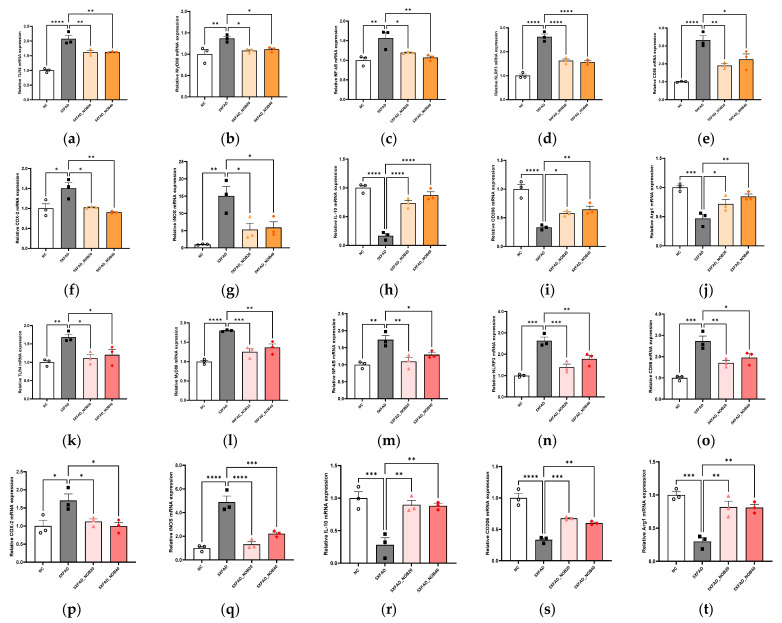
Nobiletin Modulates Microglial Activation and Inflammatory Gene Expression in the Cortex and Hippocampus of 5XFAD Mice. The mRNA expression levels of pro-inflammatory and anti-inflammatory genes in the cortical (**a**–**j**) and hippocampal (**k**–**t**) Regions were measured by RT-PCR (*n* = 3). Toll-like receptor 4 (TLR4, (**a**,**k**)); myeloid differentiation primary response 88 (MyD88, (**b**,**l**)); nuclear factor kappa B (NF-*κ*B, (**c**,**m**)); NLR family pyrin domain containing 3 (NLRP3, (**d**,**n**)); cluster of differentiation 86 (CD86, (**e**,**o**)); cyclooxygenase-2 (COX-2, (**f**,**p**)); inducible nitric oxide synthase (iNOS, (**g**,**q**)); interleukin 10 (IL-10, (**h**,**r**)); cluster of differentiation 206 (CD206, (**i**,**s**)); and arginase 1 (Arg-1, (**j**,**t**)). Mice were divided into four groups: non-transgenic control (NC), 5XFAD vehicle-treated (5XFAD), and 5XFAD mice treated orally with nobiletin at 20 mg/kg/d (5XFAD_NOB20) or 40 mg/kg/d (5XFAD_NOB40). Statistical significance was represented as * *p* < 0.05, ** *p* < 0.01, *** *p* < 0.001, and **** *p* < 0.0001; bars represent mean ± SE.

The expression levels of inflammation-related mRNA in the cortical and hippocampal regions of 5XFAD mice were analyzed to assess the activation status of the TLR4/MyD88/NF-*κ*B pathway. The results demonstrated that this inflammatory pathway was significantly activated in the 5XFAD group compared with the NC group (Figure 4a–c and Figure 4k–m).

#### 3.5.2. Pro-Inflammatory and Anti-Inflammatory Cytokines

In the 5XFAD group, this inflammatory activation was characterized by increased expression of pro-inflammatory markers, such as NLRP3, CD86, COX-2, and iNOS (Figure 4d–g and Figure 4n–q), and notable decreases in the expression of anti-inflammatory and neuroprotective markers, including IL-10, CD206, and Arg-1 (Figure 4h–j and Figure 4r–t). Conversely, in the 5XFAD_NOB20 and 5XFAD_NOB40 groups, a significant reduction was observed in the expression of these pro-inflammatory markers, indicating effective inhibition of the TLR4/MyD88/NF-*κ*B pathway (Figure 4a–g and Figure 4k–q). Additionally, the expression of anti-inflammatory and neuroprotective markers was significantly increased in the nobiletin-treated groups (Figure 4h–j and Figure 4r–t), suggesting that nobiletin exerts its therapeutic effects by suppressing inflammatory responses and promoting neuronal survival and functional recovery in the cortical and hippocampal regions of 5XFAD mice.

### 3.6. Nobiletin Increases Antioxidant Enzyme Expression Through Mitochondrial Biogenesis in the Cortical and Hippocampal Regions of 5XFAD Mice

**Figure 5:**
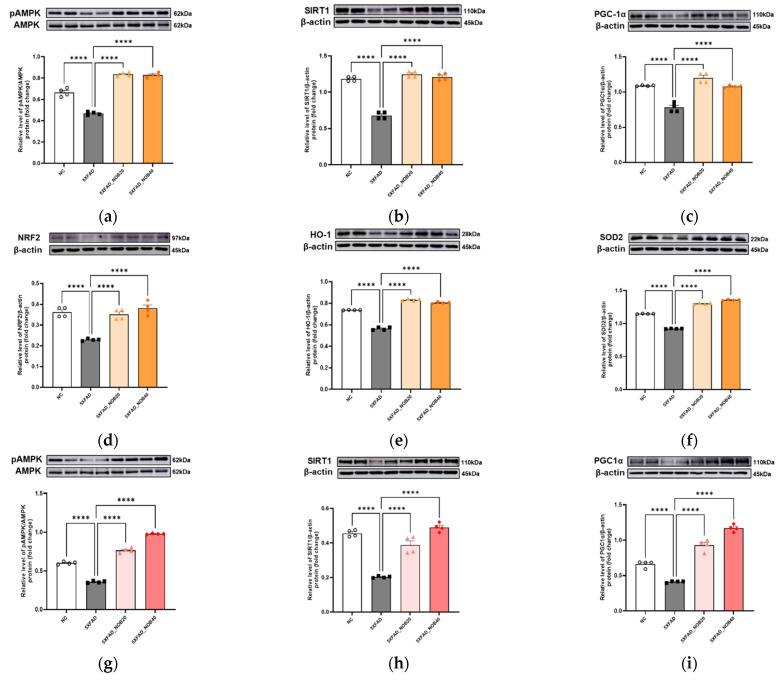

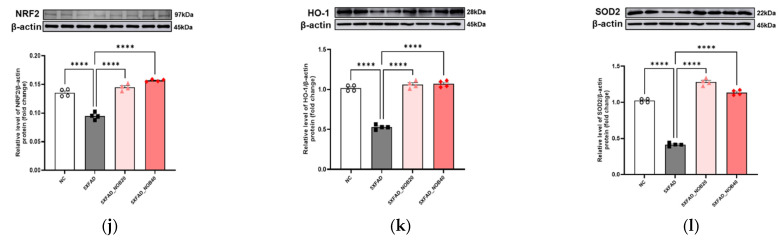
Nobiletin Activates the AMPK/SIRT1/PGC-1*α* Pathway and Enhances Antioxidant Defenses in 5XFAD Mice. Protein expression levels in the cortical (**a**–**f**) and hippocampal (**g**–**l**) regions were analyzed by Western blotting using biological replicates from individual mice (*n* = 4). Phospho-AMP-activated protein kinase (pAMPK)/AMPK (**a**,**g**); sirtuin 1 (SIRT-1, (**b**,**h**)); peroxisome proliferator-activated receptor gamma coactivator 1-alpha (PGC-1*α*, (**c**,**i**)) nuclear factor erythroid-2-related factor 2 (NRF2, (**d**,**j**)); heme oxygenase-1 (HO-1, (**e**,**k**)); superoxide dismutase 2 (SOD2, (**f**,**l**)). Nobiletin effectively restored oxidative balance and reduced neuronal oxidative stress. Mice were divided into four groups: non-transgenic control (NC), 5XFAD vehicle-treated (5XFAD), and 5XFAD mice treated orally with nobiletin at 20 mg/kg/d (5XFAD_NOB20) or 40 mg/kg/d (5XFAD_NOB40). The *β*-actin blots were derived from the same membrane after stripping and reprobing with different primary antibodies. Statistical significance was represented as **** *p* < 0.0001; bars represent mean ± SE. The upregulation of total NRF2 protein indicates increased NRF2 expression; however, NRF2 pathway activation cannot be conclusively determined because nuclear translocation was not assessed.

In the cortical and hippocampal regions of 5XFAD mice, inhibition of the AMPK/SIRT1/PGC-1*α* pathway was observed. Consistent with this finding, NRF2 expression was also significantly reduced (Figure 5a–d, *p* < 0.0001 and Figure 5g–j, *p* < 0.0001). This downregulation was associated with markedly decreased levels of the antioxidant enzymes HO-1 and SOD2 compared to those in the NC group (Figure 5e,f and Figure 5k,l). Conversely, in the nobiletin-treated groups, the AMPK/SIRT1/PGC-1*α* pathway was activated (Figure 5a–c and Figure 5g–i). This activation was associated with increased NRF2 expression and significant increases in HO-1 and SOD2 levels (Figure 5d–f, *p* < 0.0001 and Figure 5j–l, *p* < 0.0001). These findings suggested that nobiletin enhanced the oxidative stress defense system in 5XFAD mice, thereby mitigating neuronal damage and promoting functional recovery.

### 3.7. Nobiletin Promotes Neuronal Survival and Enhances Synaptic Plasticity in the Cortical and Hippocampal Regions of 5XFAD Mice

**Figure 6:**
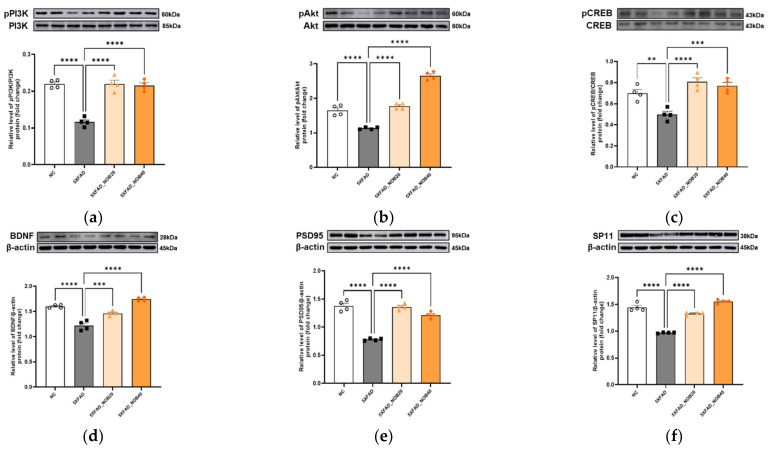

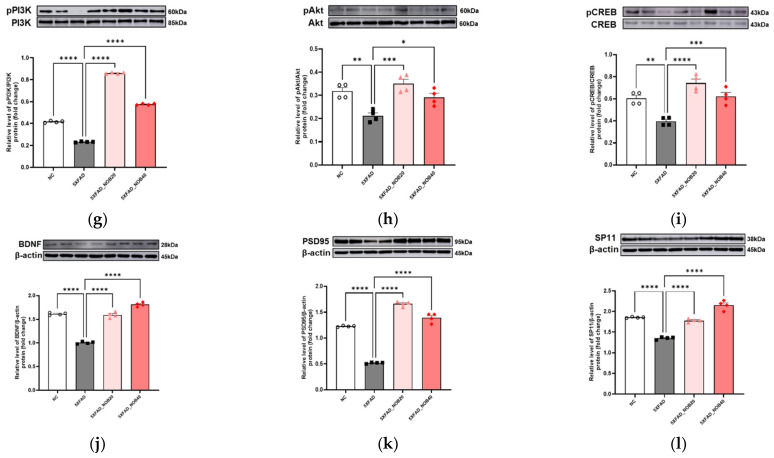
Nobiletin Increases Antioxidant Enzyme Expression through Mitochondrial Biogenesis in the Cortical and Hippocampal Regions of 5XFAD Mice. Protein expression levels in the cortical (**a**–**f**) and hippocampal (**g**–**l**) regions of the brain were analyzed by Western blotting using biological replicates from individual mice (*n* = 4). Phospho-phosphoinositide 3-kinases (pPI3K)/PI3K (**a**,**g**); phospho-protein kinase B (pAkt)/Akt (**b**,**h**); phospho-cAMP response element-binding protein (pCREB)/CREB (**c**,**i**); brain-derived neurotrophic factor (BDNF, (**d**,**j**)); postsynaptic density protein-95 (PSD95, (**e**,**k**)); synaptophysin (SP11, (**f**,**l**)). Mice were divided into four groups: non-transgenic control (NC), 5XFAD vehicle-treated (5XFAD), and 5XFAD mice treated orally with nobiletin at 20 mg/kg/d (5XFAD_NOB20) or 40 mg/kg/d (5XFAD_NOB40). The *β*-actin blots were generated on the same membrane after stripping and reprobing with different primary antibodies. Statistical significance was represented as * *p* < 0.05, ** *p* < 0.01, *** *p* < 0.001, and **** *p* < 0.0001; bars represent mean ± SE.

In the cortical and hippocampal regions of 5XFAD mice, the PI3K/Akt signaling pathway was inhibited compared to that in the NC group (Figure 6a,b and Figure 6g,h). This inhibition reduced CREB and BDNF expression (Figure 6c,d and Figure 6i,j) and significantly decreased synaptic plasticity-related factors, such as PSD95 and SP11 (Figure 6e,f, *p* < 0.0001 and Figure 6k,l, *p* < 0.0001). However, in the nobiletin-treated groups, activation of the PI3K/Akt pathway was observed (Figure 6a,b and Figure 6g,h). In line with this activation, we also found increased expression of CREB and BDNF (Figure 6c,d and Figure 6i,j), as well as a significant upregulation of PSD95 and SP11 (Figure 6e,f, *p* < 0.0001 and Figure 6k,l, *p* < 0.0001). These findings indicated that nobiletin promoted neuronal survival and enhanced synaptic plasticity in 5XFAD mice, thereby playing a crucial role in memory improvement.

## 4. Discussion

Nobiletin, a major polymethoxylated flavonoid found primarily in citrus fruits such as oranges and tangerines, has garnered significant scientific interest as a promising therapeutic candidate for neurodegenerative diseases [25,26,27]. Its diverse biological activities, including anticancer, antioxidant, anti-inflammatory, cardioprotective, and neuroprotective effects, as well as its ability to ameliorate metabolic syndromes, have been well documented [28,29,30]. Despite these promising attributes, the specific mechanisms by which nobiletin mitigates oxidative stress and inflammatory responses, enhances neuronal survival, and promotes synaptic plasticity in AD, particularly in 5XFAD mice, remain to be elucidated. Recent studies have highlighted the critical roles of oxidative stress and inflammation in the pathogenesis of AD, underscoring the importance of compounds with antioxidant and anti-inflammatory properties as therapeutic strategies. In this regard, nobiletin’s ability to activate the AMPK/SIRT1/PGC-1*α* pathway, which subsequently upregulates NRF2 and increases the expression of antioxidant enzymes such as HO-1 and SOD2 (Figure 5), suggests its potential to reinforce the brain’s oxidative stress defense system [31,32]. Nobiletin contributes to the functional recovery in AD models by reducing neuronal damage [33].

The Y-maze test results indicated that nobiletin significantly enhanced working memory in 5XFAD mice, as evidenced by a substantial increase in the spontaneous alternation rate (Figure 1a,b). This observation aligns with previous studies demonstrating nobiletin’s memory-enhancing effects, reinforcing its potential efficacy in ameliorating memory deficits associated with AD [34]. Notably, A*β* plaque accumulation is reduced following nobiletin treatment. The 5XFAD transgenic mouse model, characterized by mutations in the APP and PSEN1 genes, exhibits accelerated A*β* production and accumulation. These mice harbor five specific mutations in the APP and PSEN1 genes, resulting in increased A*β* production and plaque formation, which closely model the pathophysiology of AD [21,22]. This model is instrumental for studying disease mechanisms and evaluating the efficacy of potential therapeutic agents, such as nobiletin, in mitigating AD-related pathologies. These results demonstrate that nobiletin significantly reduced A*β* plaque levels, with the most pronounced effects observed at higher doses (5XFAD_NOB40; Figure 1d). This suggests that nobiletin effectively inhibits one of the primary pathological hallmarks of AD, thereby potentially altering disease progression.

In addition, nobiletin regulates the expression of key proteins involved in the amyloidogenic pathway. In the cortex and hippocampus of 5XFAD mice, the elevated levels of APP, BACE1, and PS1 were significantly downregulated after nobiletin treatment (Figure 3). APP is cleaved by BACE1 and PS1 to generate A*β* peptides [35]. Conversely, ADAM10, which functions as an *α*-secretase, cleaves APP to promote the non-amyloidogenic pathway, thus preventing A*β* formation [36,37]. The upregulation of ADAM10 in the nobiletin-treated groups supports a shift toward non-amyloidogenic APP processing. Collectively, these findings indicate that nobiletin suppresses the amyloidogenic pathway while enhancing the non-amyloidogenic pathway, potentially delaying the progression of AD. Increased inflammatory responses and oxidative stress are pivotal for the progression and exacerbation of AD symptoms [38]. Pro-inflammatory cytokines such as IL-6, IL-1*β*, and TNF-*α* contribute significantly to neuronal damage [39]. Nobiletin effectively mitigates these pathological processes [27,40,41]. This study revealed that nobiletin treatment decreased serum levels of proinflammatory cytokines and increased antioxidant enzyme levels, including SOD, CAT, and GSH-Px, in 5XFAD mice (Figure 2). Therefore, nobiletin effectively alleviates inflammation and oxidative stress, critical factors in AD progression, and may also contribute to neuronal protection and disease modulation.

Furthermore, in the cerebral cortex and hippocampus, nobiletin inhibited the activation of the TLR4/MyD88/NF-*κ*B pathway, accompanied by reduced pro-inflammatory indices and elevated anti-inflammatory and neuroprotective indices. Neuroinflammation is a significant feature of AD, with activation of the TLR4 and MyD88 pathways that induce inflammatory responses through NF-*κ*B activation [41]. Nobiletin suppresses neuroinflammation, like irisin, by inhibiting TLR4 and MyD88 activation and preventing NF-*κ*B pathway activation, thereby inhibiting NLRP3 inflammasome activation and reducing pro-inflammatory cytokines while increasing anti-inflammatory cytokines [40]. This suppression promotes neuronal survival and functional recovery.

Moreover, nobiletin enhances antioxidant enzyme expression through mitochondrial biogenesis, which is mediated by the AMPK/SIRT1/PGC-1*α* pathway. AMPK regulates the SIRT1/PGC-1*α*-dependent antioxidant system, which is crucial for maintaining mitochondrial homeostasis and responding to oxidative stress [42]. The transcription factor NRF2, a key regulator of the oxidative stress response, promotes the expression of antioxidant enzymes such as HO-1 and SOD2 [43]. The subsequent increase in NRF2 expression, along with the elevated levels of these antioxidant enzymes, suggests that nobiletin strengthens oxidative stress defense mechanisms, thereby protecting neurons and aiding in functional recovery. These findings are consistent with a possible enhancement of NRF2-related antioxidant signaling but, by themselves, do not demonstrate definitive NRF2 pathway activation. The multifaceted actions of nobiletin underscore its potential as a therapeutic agent for AD, highlighting its capacity to modulate key pathways involved in neuroinflammation and oxidative stress.

Finally, nobiletin promoted neuronal survival and synaptic plasticity. In parallel, our results showed that nobiletin activated the PI3K/Akt pathway and increased the expression of CREB, BDNF, and synaptic plasticity-related markers PSD95 and SP11 in the cortex and hippocampus. The PI3K/Akt pathway is a crucial signaling pathway involved in cell survival, growth, metabolism, migration, and proliferation, and promotes CREB activation [44]. Activated CREB translocates to the nucleus to facilitate the expression of BDNF [45], increasing the expression of synaptic plasticity-related factors, such as PSD95 and SP11 [46,47]. These findings suggested that nobiletin enhanced memory performance by promoting neuronal survival and synaptic plasticity.

In summary, nobiletin exhibits multiple beneficial effects in the 5XFAD mouse model of AD, including reduced A*β* plaque accumulation, modulation of amyloidogenic pathways, suppression of neuroinflammation, enhancement of oxidative stress defenses, and promotion of neuronal survival and synaptic plasticity. These multifaceted actions underscore the therapeutic potential of nobiletin in AD and warrant further investigation to fully elucidate its mechanisms of action and optimize its application in clinical settings.

Unlike previous studies that primarily focused on nobiletin’s antioxidant and anti-inflammatory effects, the present study provides novel mechanistic insights into how nobiletin enhances synaptic plasticity in the context of Alzheimer’s disease. Specifically, this work is the first to demonstrate, in a 5XFAD transgenic mouse model, that nobiletin promotes neuronal survival and cognitive improvement by activating the PI3K/Akt–CREB–BDNF signaling pathway. This mechanistic elucidation distinguishes our study from earlier reports, which primarily emphasized the compound’s general neuroprotective or anti-amyloid properties without addressing its direct influence on synaptic plasticity-related signaling cascades.

## 5. Conclusions

This study demonstrates that nobiletin exerts significant neuroprotective effects in a 5XFAD mouse model of AD by attenuating oxidative stress and neuroinflammation. These effects are associated with improved synaptic plasticity, which stems from nobiletin’s ability to mitigate key pathological processes. Furthermore, nobiletin enhances neuronal survival, modulates amyloidogenic pathways, and strengthens the brain’s intrinsic antioxidant defenses. These findings highlight the multifaceted therapeutic potential of nobiletin for the treatment of AD and other neurodegenerative disorders, warranting further investigation of its clinical applications.

Despite these promising findings, this study has several limitations. First, only male 5XFAD mice were included, and sex-specific differences were not examined. Second, the treatment period was limited to 4 weeks, and longer-term studies are needed to confirm the sustained efficacy and safety of nobiletin. Third, although antioxidant enzyme activities were assessed in serum and related signaling pathways were analyzed in brain tissue, direct measurements of antioxidant enzyme activities in the brain were not conducted. In addition, downstream targets beyond the PI3K/Akt–CREB–BDNF axis were not fully explored. Finally, long-term toxicity and potential tolerance to nobiletin were not evaluated. Further studies addressing these limitations will help to better define the therapeutic potential and underlying mechanisms of nobiletin in Alzheimer’s disease.

## Data Availability

The data supporting the findings of this study are available from the corresponding author upon request.
